# Gender Perspectives on the Global Burden of Dementia: The Impact of Metabolic and Behavioral Risk Factors from 1990 to 2021 and Projections for 2036—A Systematic Analysis Using the GBD Database

**DOI:** 10.1002/alz70860_100695

**Published:** 2025-12-23

**Authors:** Fangshuo Cheng, Fen Sun

**Affiliations:** ^1^ College of Basic Medicine, Hangzhou Normal University, Hangzhou, Zhejiang, China; ^2^ College of Basic Medicine, Hangzhou Normal University, Hangzhou, Zhejiang Province, China; ^3^ Department of Neurology, The Affiliated Hospital of Hangzhou Normal University, Hangzhou, Zhejiang, China

## Abstract

**Background:**

Alzheimer's disease (AD) is an irreversible, age‐related condition. ^[1,2]^Studies have shown that the prevalence and mortality of AD and other dementias (ADOD) are approximately 1.73 times higher in women than in men. ^[3,4]^Additionally, the global burden of dementia attributable to metabolic risk factors is increasing, with significant gender differences in their impact on dementia. ^[4]^The gender differences in the burden of all‐cause mortality of ADOD have yet to be systematically explored.

**Method:**

The disease burden data was derived from the Global Burden of Disease Study (GBD) 2021, describing the distribution characteristics of all‐cause mortality of ADOD attributable to specific risk factors across multiple dimensions. The study employed various methods to quantify trends and project future changes, constructing a “frontier” analysis.

**Result:**

From 1990 to 2021, the disease burden attributable to metabolic factors increased, with the burden of high body mass index (HBMI) rising approximately twice as fast in men compared with women. The burden of high fasting plasma glucose (HFPG) increased at similar rates in both sexes. The decline in smoking‐attributable all‐cause mortality of ADOD was approximately twice as fast in women compared with men. By 2036, the all‐cause age‐standardized mortality rate (ASMR) of ADOD in men aged 40 and above is projected to reach 17.13, with HFPG as the largest risk factor and HBMI as the fastest‐growing risk factor. In women, the ASMR of ADOD is projected to reach 19.98, with HFPG as both the largest and fastest‐growing risk factor. Frontier analysis showed that the greatest potential for reducing the burden of HBMI lies in high SDI countries, HFPG in low‐middle SDI countries, and smoking in high‐middle SDI countries.

**Conclusion:**

The growing impact of metabolic risk factors underscores urgent global health challenges, with gender‐specific trends highlighting disparities. Targeted interventions addressing HBMI, HFPG, and smoking in specific SDI regions are critical for effective prevention.

